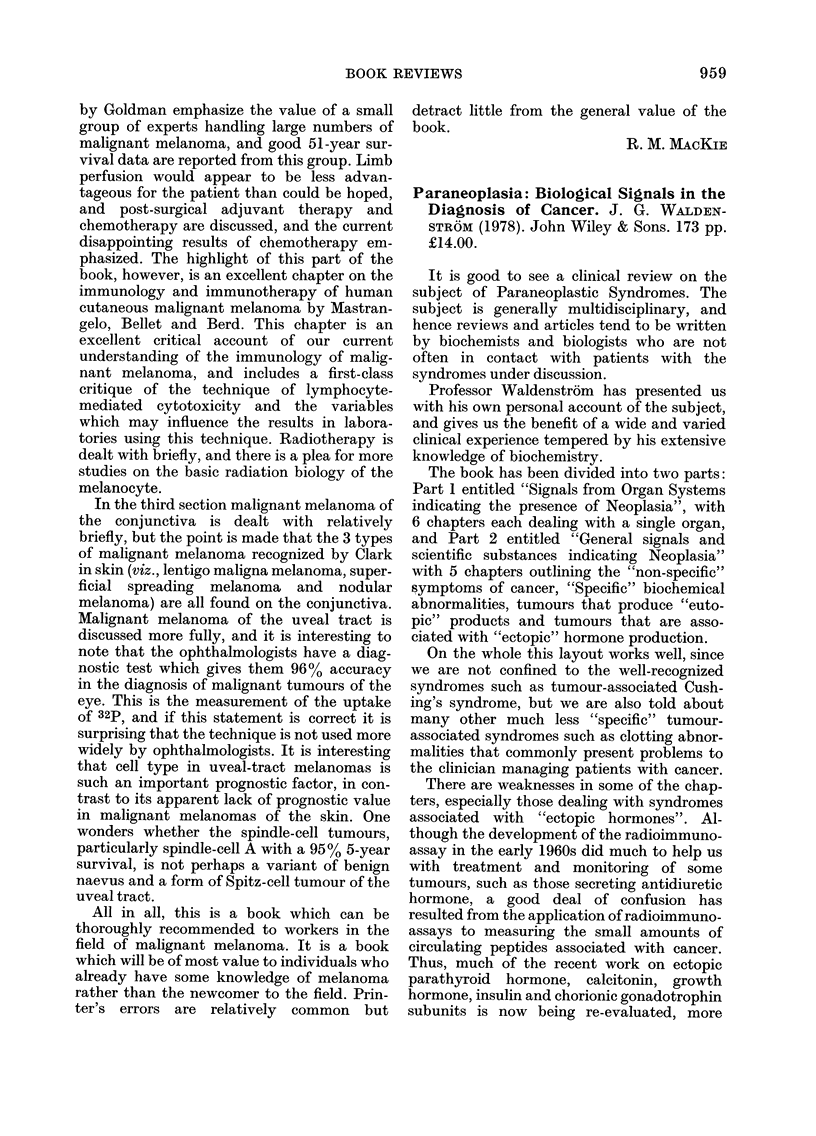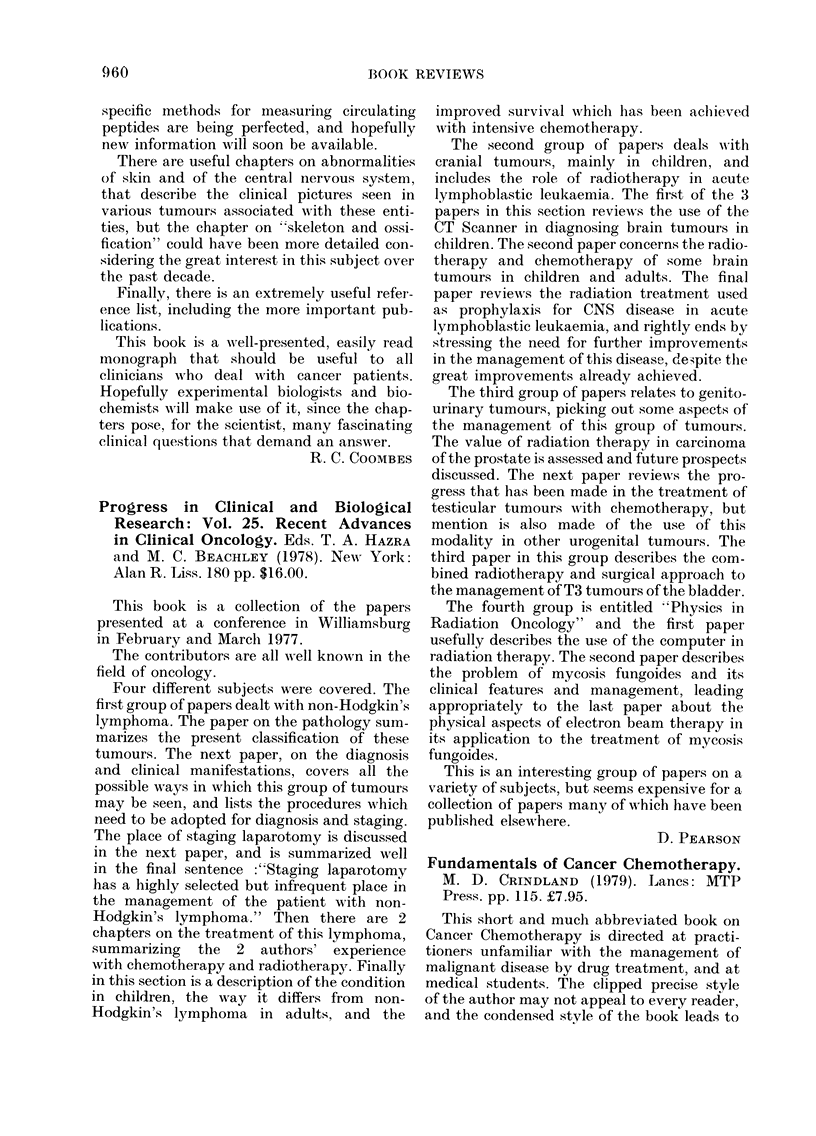# Paraneoplasia: Biological Signals in the Diagnosis of Cancer

**Published:** 1979-12

**Authors:** R. C. Coombes


**Paraneoplasia: Biological Signals in the Diagnosis of Cancer.** J. G. Waldenström (1978). John Wiley & Sons. 173 pp. £14.00.

It is good to see a clinical review on the subject of Paraneoplastic Syndromes. The subject is generally multidisciplinary, and hence reviews and articles tend to be written by biochemists and biologists who are not often in contact with patients with the syndromes under discussion.

Professor Waldenström has presented us with his own personal account of the subject, and gives us the benefit of a wide and varied clinical experience tempered by his extensive knowledge of biochemistry.

The book has been divided into two parts: Part 1 entitled "Signals from Organ Systems indicating the presence of Neoplasia", with 6 chapters each dealing with a single organ, and Part 2 entitled "General signals and scientific substances indicating Neoplasia" with 5 chapters outlining the "non-specific" symptoms of cancer, "Specific" biochemical abnormalities, tumours that produce "eutopic" products and tumours that are associated with "ectopic" hormone production.

On the whole this layout works well, since we are not confined to the well-recognized syndromes such as tumour-associated Cushing's syndrome, but we are also told about many other much less "specific" tumour-associated syndromes such as clotting abnormalities that commonly present problems to the clinician managing patients with cancer.

There are weaknesses in some of the chapters, especially those dealing with syndromes associated with "ectopic hormones". Although the development of the radioimmunoassay in the early 1960s did much to help us with treatment and monitoring of some tumours, such as those secreting antidiuretic hormone, a good deal of confusion has resulted from the application of radioimmunoassays to measuring the small amounts of circulating peptides associated with cancer. Thus, much of the recent work on ectopic parathyroid hormone, calcitonin, growth hormone, insulin and chorionic gonadotrophin subunits is now being re-evaluated, more specific methods for measuring circulating peptides are being perfected, and hopefully new information will soon be available.

There are useful chapters on abnormalities of skin and of the central nervous system, that describe the clinical pictures seen in various tumours associated with these entities, but the chapter on "skeleton and ossification" could have been more detailed considering the great interest in this subject over the past decade.

Finally, there is an extremely useful reference list, including the more important publications.

This book is a well-presented, easily read monograph that should be useful to all clinicians who deal with cancer patients. Hopefully experimental biologists and biochemists will make use of it, since the chapters pose, for the scientist, many fascinating clinical questions that demand an answer.

R. C. COOMBES